# The influence of meaningful work on the mental health of SME employees in the COVID-19 era: can coping strategies mediate the relationship?

**DOI:** 10.1186/s12889-023-17347-3

**Published:** 2023-12-06

**Authors:** Muhammad Farhan Jalil, Azlan Ali

**Affiliations:** 1 Faculty of Economics and Business, Universiti Malaysia Sarawak (UNIMAS), 94300 Kota Samarahan, Sarawak, Malaysia; 2 Graduate School of Management, Management and Science University, Shah Alam, Selangor Darul Ehsan Malaysia

**Keywords:** Meaningful work, Coping strategies, Mental health, SMEs, Structure equation modelling, COVID-19

## Abstract

**Background:**

Stress, depression, and anxiety are prevalent issues among SME employees during the COVID-19 pandemic. Even while having meaningful work that expressively contributes to individual growth has been related to improving mental health, employees’ work may also need to adopt coping strategies to increase outcomes. The purpose of this study was to examine the relationship between meaningful work (positive meaning, meaning-making, and greater good motivations) and mental health, as well as coping strategies (problem-focused and emotion-focused) as a mediator of this relationship.

**Methods:**

Meaningful work, coping strategies, and mental health were evaluated in empirical research based on a sample of 462 SME employees working in Malaysia. Structured questionnaires were used to collect the data and analyze it through Structural Equation Modelling (SEM) using AMOS 21.0.

**Results:**

The findings of the study show the importance of meaningful work in influencing the mental health of SME employees, particularly during a crisis like the COVID-19 pandemic. This suggests that the more they value and see their work as meaningful, the more capable they are of dealing with limitations and mental health problems associated with crises. The study also discovered a partial mediating role for coping strategies between employees’ mental health and meaningful work.

**Conclusion:**

This study encourages employees to constantly feel connected and discover continued possibilities to work and learn even during crisis situations. In order to improve human resource efficiency in emerging markets, managers and owners of SMEs must implement the model developed by the researchers.

## Introduction

Over the last twenty years, organizational psychology research has switched from unpleasant work experiences to employee well-being [1], with a rising emphasis on meaningful work for individuals and businesses. Goh and Baum [2] link positive work behavior and attitudes to meaningful work. Chen et al. [3] highlight that employees consider meaningful work more meaningful and vital than economic rewards and employment security. Li et al. [4] noted that in today’s business landscape, an increasing number of employees place significant importance on their own feelings. This observation highlights the growing emphasis on meaningful work in the workplace. As a result, work that holds meaning and positively contributes to the well-being of others is increasingly cherished within organizational settings.

May et al. [5] stated that “meaningful work involves physical well-being, complicated work that allows for growth and self-expression, emotional involvement, and financial stability” (p. 652). Furthermore, according to Hackman and Oldham [6], “meaningful work is the degree to which the employee views the job as generally significant, useful, and worthwhile” (p. 161). Bailey et al. [7] consider deliberate meaningful work to be a subcategory of the desire for self-actualization. Mousa and Samara [8] say meaningful work satisfies an innate need for employees to experience more satisfaction in their jobs, flourish in the face of adversity, maintain positive connections with coworkers, and attain better performance levels. Meaningful work can also increase employee engagement, which is linked to higher productivity, creativity, and morale [9]. It is essential for employers to create an environment in which employees can find meaning in their work and feel empowered to do their best work.

Despite the increasing body of studies on the matter, little research has used meaningful work as an interpreter of employees’ psychological well-being. This seems to be regrettable as mental health represents a feeling of internal balance that allows workers to perform their social obligations and deal with society’s obstacles [10], which are becoming increasingly complicated. In essence, mental health is one of the United Nations’ (UN) sustainable development goals that member countries are encouraged to attain and secure [11], since it inspires people to pursue wellbeing and success in both their personal and professional lives [8]. Changes in job environments may bring a fear of unemployment, a struggle to deal with new workplaces, and difficulty maintaining their social status, which can lead to depression or anxiety-related disorders [12]. These are all considered clear indications of poor mental health. In emerging markets like Malaysia, the unfavorable symptoms are seen as the primary reason for filing for sick leave [13]. Furthermore, they pose a danger to employees’ well-being and perceived self-respect.

Mental health and the meaningfulness (or lack thereof) of work are important factors that can significantly impact Malaysia’s growth and development [14]. According to Junça-Silva et al. [15], mental health and meaningful work are closely tied to employee productivity and engagement. When employees experience good mental health and find their work meaningful, they are more likely to be engaged, motivated, and productive. This, in turn, can lead to increased efficiency and higher output, contributing positively to Malaysia’s economic growth. According to Rahman et al. [16], employees who are mentally well and find their work meaningful are often more innovative and creative. They come up with new ideas and solutions, which can drive innovation within organizations and across industries. Innovation is a key driver of economic growth in the modern global economy. Furthermore, according to Penninx et al. [17], addressing mental health concerns and promoting meaningful work can result in reduced healthcare costs for individuals and organizations. Healthier employees are less likely to require extensive medical treatment [18], thus lowering the burden on the healthcare system and allowing for more productive workforces. Abdul Jalil et al. [19] described that mental health and meaningful work also contribute to the overall well-being of Malaysians. When people are mentally healthy and engaged in work, they find meaningful, they tend to lead more fulfilling lives [20]. This can lead to reduced social issues, better quality of life, and a more stable and contented society. To promote Malaysia’s growth, it is essential for the government, employers, and society at large to prioritize mental health and create workplaces that offer meaningful work experiences [21]. This includes addressing mental health stigma, providing mental health support services, and ensuring that jobs are designed to be engaging and purposeful. A mentally healthy and engaged workforce is a valuable asset for any nation’s economic growth and development [22].

Small and Medium Enterprises (SMEs) play a crucial role in the success of Malaysia. According to Laila et al. [23], SMEs make up a significant portion of Malaysia’s economy, contributing substantially to its Gross Domestic Product (GDP). They are key drivers of economic growth, job creation, and innovation. Au et al. [24] explained that, SMEs are major employers in Malaysia, providing jobs for a substantial portion of the workforce. Their ability to create employment opportunities helps reduce unemployment rates and enhances the overall well-being of the population. Wasiuzzaman et al. [25] stated that SMEs are dispersed across different regions in Malaysia, including rural areas. Their presence helps reduce regional disparities in terms of economic development and promotes more inclusive growth. Cheong et al. [26] described that SMEs are often more agile and innovative than larger corporations. They can experiment with new ideas and technologies, fostering entrepreneurship and contributing to Malaysia’s competitiveness on a global scale. Andriamahery and Qamruzzaman [27] describe that SMEs empower individuals, including women and marginalized groups, to become entrepreneurs and contribute to society. They promote social development and reduce income inequality. Overall, SMEs in Malaysia are essential because they drive economic growth, create jobs, foster innovation, support regional development, and contribute to the overall well-being of the country. Their dynamism and adaptability make them vital contributors to Malaysia’s success.

The research by Martin et al. [28] underscores a significant gap in SMEs regarding mental health policies. It found that less than 25% of SME managers or owners had clear policies on mental health. However, interestingly, they acknowledged that discussing topics like depression in the workplace was appropriate. This suggests a need for greater attention to mental health support in SMEs.

Lindström’s [29] suggestion that SMEs require special attention in mental health interventions is well-founded. SMEs often have limited resources, both in terms of knowledge and finances, to implement comprehensive mental health programs [30]. Stress management, mental health literacy, and employee support programs are effective strategies commonly used in larger corporate settings [31]. However, these strategies can be challenging to implement and are seldom utilized by SME owners or managers, as noted by De Angelis et al. [32]. The limitations of resources and expertise in SMEs can make it difficult to execute such programs effectively. Small businesses, despite their resource constraints, can offer a conducive environment for promoting employee well-being [33]. Addressing mental health in SMEs is crucial, as it not only benefits individual employees but can also contribute to the overall success and growth of these businesses [32].

Since its initial detection in China in November 2019, coronavirus disease-2019 (COVID-19) has caused more than 514 million infections and 6.3 million deaths worldwide [34]. Furthermore, this rapidly transmitted virus has prompted the globe to impose restriction measures [35] and also cease many typical business and job activities due to its symptoms of diarrhea, persistent cough, and high temperature [36]. The worldwide COVID-19 pandemic has created a global atmosphere of despair and stress [37], which is followed by concerns of financial loss, employment uncertainty, and dread of the future [38]. This demonstrates the harmful impact on people’s mental health. Small and medium enterprise (SME) employees were not exempt from the negative externalities of the pandemic since they were forced to relocate their jobs to a work-from-home environment [39]. Employees, in other words, were forced to mourn their prior working circumstances while abruptly transitioning to a new system in an environment of uncertainty.

The research on disaster management highlights the different environmental challenges that individuals face during a crisis. To adjust, employees must create new tactics [40]. The former focuses on controlling emotions to make a difficult situation more tolerable, whereas Lazarus [41] emphasizes active coping strategies to deal with the stressful situation. Coping strategies can also have a substantial psychological impact on coping outcomes [42]. According to Lazarus and Folkman [43], “problem-focused coping” and “emotion-focused coping” are two types of coping strategies. According to current research (such as [28; 44; 45; 46], coping strategies are beneficial in lowering stress and improving the mental health of employees in SMEs in developing markets. The research of Chen et al. [47] has found that coping strategies are effective in lowering stress and improving employees’ mental health in times of crisis.

Due to the expansion of COVID-19 and the fact that no previous research has empirically understood the relationship among meaningful work, coping strategies, and mental health. Based on previous studies, the goal of this study is to determine the possible influence of meaningful work in affecting the mental health of Malaysian SME employees and the mediating role of coping strategies in the relationship. The following research questions are addressed in this study:

*RQ1: “Does meaningful work influence the mental health of SME employees in Malaysia during the COVID-19 pandemic?”*

*RQ2: “In Malaysia during the COVID-19 pandemic, do coping strategies mediate the relationship between meaningful work and employee mental health?”*

This research, which focuses on SME employees who work in the world’s most uncertain and unpredictable working environment, suggests an effort to assess the problem and suggest practical solutions to assist these individuals.

Furthermore, the study significantly contributes to both practice and theory. Theoretically, it contributes to self-determination theory and also has a prospective influence on employee mental health, in addition to addressing a relevant and crucial understudied problem. SME employees must keep a sense of understanding and continue to work, learn, and enhance their capabilities in order to preserve a perception of importance in their work responsibilities amid a crisis and, as a result, reduce any mental health difficulties. This research has managerial implications: Initially, for SME owners or managers, it emphasizes the significance of giving meaningful work to employees, particularly during times of crisis. Furthermore, it proposes that employees should consider seeing psychiatrists or counsellors on a constant schedule to check their levels of anxiety and distress, particularly during times of crisis and massive change.

## Literature review

### Self-determination theory

Ryan and Deci [48] recommend the self-determination theory (SDT), which focuses on human motivation and shows how individuals dedicate time and effort to activities that help them cope with mental health issues. Furthermore, Ryan and Deci [48] explain that SDT is relevant to mental-health research since it exclusively concentrates on human motivation by claiming that individuals have an inbuilt ability for personal development under suitable circumstances. The theory takes into account the individual’s drive to select and make decisions, as well as feelings of competence and affiliation [49]. Individuals who meet these psychological requirements are more likely to work hard and feel at ease at work [8]. This theory has been used to explain why some employees are successful in their roles while others struggle. It is also a useful tool for managers to assess the motivations and needs of their teams.

Meaningful work and mental health are closely related to SDT [50]. It examines individuals’ innate psychological needs for autonomy, competence, and relatedness in order to understand their motivation and well-being [48]. SDT focuses heavily on autonomy, which is the notion that individuals need to feel in control of their behavior. Employees can match their tasks with their personal values and interests when granted autonomy in their roles within meaningful work [51]. This feeling of independence helps people see their work as significant, which improves job satisfaction and psychological health [8]. According to Scarduzio et al. [52], SDT also emphasizes competence, which includes feeling capable and effective in activities. Furthermore, they explain that when employees engage in meaningful work that uses their strengths and skills, they feel competent. This sense of mastery and accomplishment has a positive impact on their mental health and self-esteem. Ntoumanis et al. [49] explain that social connections and relatedness play an important role in SDT. It is often necessary to collaborate, work together, and make a positive impact on others in order to do meaningful work. A sense of connectedness to colleagues and the larger purpose of work leads to greater job satisfaction and well-being for individuals [48].

Ntoumanis et al. [49] explain that SDT related with the maintaining mental health and overall wellbeing depends on addressing the fundamental psychological requirements of autonomy, competence, and relatedness. People are more likely to have favorable mental health outcomes, such as less stress, increased resilience, and improved emotional wellbeing. This is when they have self-determination opportunities at work and feel competent and connected to others [51]. Overall, SDT provides a comprehensive framework for understanding the connections between meaningful work, mental health, and employee wellbeing [52]. Organizations may build work environments that encourage meaningful work experiences and promote excellent mental health outcomes among their workers. This is done by fulfilling people’s fundamental psychological needs for autonomy, competence, and relatedness [51].

### Meaningful work

The concept of meaningful work, as described by Asik-Dizdar and Esen [53], is rooted in the idea that there is a positive relationship between an individual’s level of engagement in their work and the sense of fulfillment and satisfaction they derive from it. These outcomes encompass a range of aspects including happiness, effectiveness, and contentment, among others, which collectively contribute to a sense of intrinsic value in the work an individual performs (p. 5).

May et al. [54] offer a complementary definition, defining meaningful work as the inherent value of a job’s purpose or objectives, as evaluated against an individual’s personal beliefs or ideals. This perspective emphasizes the alignment between an individual’s values and the goals of their work, highlighting the significance of finding purpose and fulfillment in one’s professional endeavors (p. 14).

Steger et al. [55] view meaningful work as a reflection of a job’s importance and how specific activities and responsibilities are evaluated. Individual and professional goals are more likely to be attained by employees who have a sense of purpose at work [8]. According to Bailey and Madden [56], meaningful work enables individuals to grasp not just their profession’s purpose but also their place in society. This helps to explain the increasing application of this notion in many professional settings [57].

Recent research has found a link between meaningful work and significant organizational outcomes, including psychological well-being [58], job satisfaction [59], and organizational commitment [60]. According to Jung and Yoon [61], there is a link between the meaningfulness of work and the affective commitment of employees to their enterprise. Furthermore, the research also reveals a link between meaningful work and the performance of an employee. The study of Mousa and Samara [8] finds that there is little empirical research on the importance of meaningful work for vocational psychology.

According to Mousa and Samara [8], job responsibilities, worker duty, relationships with co-workers, and contacts with outside stakeholders are the four sources of meaningful work. Similarly, according to Wang and Xu [62], the worker, stakeholders, the firm, and spiritual life are all elements of meaningful work. According to Jabeen et al. [63], the life experiences of employees influence their perception of the value of a certain job. In their study, Steger et al. [55] identified three aspects of meaningful work: “the first is positive meaning, which represents how important an employee feels his or her job is; the second is meaning making, which measures how much an employee feels his job is just one part of a greater picture in people’s lives; and the third is greater good motivation, which indicates the employee’s beneficial impact on society, individuals, and maybe humanity” (p. 5). Indeed, such meaningful beliefs can aid in the treatment of some of the increasingly prevalent mental health issues, particularly during crisis situations.

### Mental health

In recent decades, there has been a notable increase in the prevalence of mental illnesses. Research by Kessler et al. [64] suggests that a significant portion of the global population, at least 18%, may encounter mental health issues at some point in their lives. Psychiatric researchers have extensively studied the challenges faced by individuals regarding their mental health. As noted by Kotera et al. [65], poor mental health can give rise to feelings of guilt and can hinder a person’s ability to take care of themselves or attend to the needs of others. The impact of mental health difficulties goes beyond the individual, affecting their relationships and overall quality of life. Recognizing and addressing mental health concerns has become increasingly important in today’s society to support individuals in achieving and maintaining good mental well-being.

According to Muris [66], self-compassion can be defined as the recognition that mental illnesses are part of the human experience, leading to a sense of empathy for both oneself and others who struggle with mental health challenges. It emphasizes understanding and kindness toward oneself and those facing mental illnesses. The study by Brouwers [67] highlights that individuals dealing with mental health issues may experience reduced efficiency and decreased interaction with their colleagues. This can have implications for their workplace performance and relationships.

Similarly, Joshi and Sharma [68] point out that individuals grappling with mental difficulties may be more susceptible to a diminished sense of self-worth and a decreased sense of belonging. Mental health challenges can affect one’s self-esteem and social connections. Furthermore, as noted by Yang et al. [69], individuals facing mental health issues may encounter ostracism and isolation in various business and personal settings. This social isolation can exacerbate the challenges associated with mental health. It’s worth mentioning that poor mental health often manifests in symptoms such as sleep disturbances, as highlighted by Muris [66], and a lack of self-compassion. Recognizing and addressing these symptoms is essential for promoting mental well-being and fostering a more supportive and inclusive environment for individuals facing mental health challenges.

In many developing countries, including Malaysia, there has been limited acknowledgment of the risks associated with mental health issues, as highlighted by Yan et al. [37]. However, there have been some positive developments in Malaysia’s approach to mental health; First - Government Initiatives: The Malaysian government has taken steps to address mental health concerns. It has introduced a strategic psychiatric policy aimed at improving access to mental healthcare, endorsing therapies for psychiatric disorders, and training competent professionals and educators to handle mental health issues [70]. Second - Research Efforts: Research institutes have been established to conduct empirical studies on methods to alleviate and create a more resourceful framework for addressing psychological illnesses, as noted by Mousa & Samara [8]. This research is vital for developing effective strategies and interventions. However, as emphasized by Torous et al. [71] and Uzir et al. [72], it remains crucial to ensure that mental health support reaches those who need it, especially in the context of SMEs in underdeveloped countries, where mental health issues are often overlooked. In such situations, it becomes essential to explore ways to prevent mental health problems, particularly when access to psychiatric therapy and mental health knowledge is limited, and there is a lack of attention from governments and businesses.

Addressing mental health issues requires a multifaceted approach, including awareness campaigns, training programs, and accessible support systems. It’s a global challenge that calls for collaboration between governments, organizations, healthcare providers, and communities to create a more inclusive and supportive environment for individuals facing mental health difficulties.

### Coping strategies

Lazarus and Folkman [43] established the Transaction Model of Coping, which defines it as “the process of a potentially stressful person-environment transaction.“ Their approach included individual and ecological influencing factors, coping, cognitive evaluation, stress, and consequences. Furthermore, Taylor and Schneider [73] claim that cognitive assessment and coping are important predictors of traumatic individual interactions and their immediate and long-term consequences. The transactional model, according to Lazarus and Folkman [74], explains that stress or anxiety is a relationship between a person’s environmental expectations and the accessibility of resources to react.

Furthermore, Lazarus and Folkman [43] define “coping” as a fundamental approach for managing stress or anxiety, encompassing ever-evolving psychological and behavioral methods employed to navigate challenging personal situations. Within this framework, Lazarus and Folkman [43] distinguish between two primary coping strategies: problem-focused and emotion-focused.

- *Problem-focused coping*: This strategy is geared towards addressing stressors by taking actions to avoid, create distance from, or selectively attend to them. Its primary aim is to reduce or manage the emotional distress associated with the stressors by addressing the root causes or challenges.
- *Emotion-focused coping*: In contrast, this strategy focuses on regulating emotional responses to stressors by employing methods such as avoidance, distancing, or selective attention. The goal is to mitigate or manage the emotional distress associated with stressors without necessarily addressing their underlying causes.

Prayag et al. [75] elaborate that both problem-focused and emotion-focused coping strategies can serve as immediate responses to stress, offering individuals ways to navigate and adapt to stressful situations.

According to a recent study by Jalil et al. [40], being emotion-focused might be more influential in situations where a person has less control over anxiety and/or distress. Prayag et al. [75] mention that evaluating coping effectiveness necessitates a scrutiny of the situation or environment. Employee coping mechanisms in relation to stress and emotions at work and how to deal with the potential repercussions of business success and growth have been the subject of SME literature. However, none of the research examines COVID-19 in the context of a pandemic.

## Hypotheses development

### Meaningful work and mental health

According to Lysova et al. [76], employment that is meaningful, promotes individual achievement, and advances society is referred to as “meaningful work.“ Numerous studies of career development in vocational psychology [such as 77; 78; 79] have offered determinants of job satisfaction, but meaningful work has been largely ignored. According to Soomro et al. [80], contentment emerges when a company is able to meet the attitudes, objectives, and demands of its employees. Furthermore, according to Lent and Brown [81], self-efficacy, positive affect, working circumstances, and goal development all predict satisfaction both intrinsically and extrinsically. However, Mousa and Samara [8] argue that although meaningful work is a vital element of psychological well-being at the workplace, it is not expressly included in these models. According to Wehmeyer et al. [82], a career development model called “career building” has addressed the problem of meaning. Career building is a narrative therapy strategy that teaches counsellors how clients build and enforce meaning in their lives rather than the perceived relevance and importance of someone’s job [83]. Thus, the field of vocational psychology has to continue to produce meaningful work.

Steger et al. [55] address this by pinpointing the fundamental elements of meaningful work and suggesting a measurement approach that closely mirrors these attributes. They define meaningful work as encompassing three key components, emphasizing the necessity of incorporating all these aspects in any subsequent research on the construct.

#### Positive meaning in work

The concept of “positive meaning in work” stands as a pivotal element within the framework of meaningful work [55]. According to Allan et al. [84], when individuals derive positive meaning from their work, it indicates that they perceive their tasks and contributions as valuable, purposeful, and personally fulfilling. Meaningful work frequently aligns with an individual’s values, interests, and passions [76]. When employees find their work personally gratifying, it fosters a positive sense of meaning. As highlighted by Sinsky et al. [85], the belief that one’s work is meaningful and valued can play a role in reducing stress levels. The sense of achievement and purpose can serve as a protective barrier against the adverse impacts of workplace stress. According to Allan et al. [83], individuals who discover positive meaning in their work are more likely to experience job satisfaction. Job satisfaction is intricately linked with mental well-being [86], considering the substantial time individuals dedicate to their work. Those who find positive meaning in their work often exhibit greater resilience in the face of challenges. The conviction that their work serves a larger purpose aids individual in coping with setbacks and difficulties, fostering mental resilience. Munn [87] identified that identifying positive meaning in work often involves recognizing the significance of maintaining a healthy work-life balance. Organizations prioritizing meaningful work often endorse policies that contribute to a balanced lifestyle, thereby diminishing the risk of burnout and mental health issues [88]. Drawing from the existing literature, positive meaning in work emerges as a pivotal component of meaningful work that encompasses and promotes mental health. Small and medium-sized enterprises (SMEs) that prioritize positive meaning in work are likely to make substantial contributions to the overall well-being of their workforce. Consequently, we posit the following hypotheses:

• *Hypothesis 1a. Positive meaning in work is significantly related to meaningful work.*

#### Meaning making through work

Deriving meaning through work is a foundational element of meaningful work and can significantly contribute to enhanced mental health [84]. The process of extracting meaning from one’s work involves discovering purpose, significance, and a sense of accomplishment in the tasks and contributions undertaken [76]. According to Scott [89], meaning-making through work imparts a sense of purpose to individuals. Connecting daily tasks and responsibilities to a broader purpose or goal enhances their overall sense of meaning and direction in life. As highlighted by Van den Heuvel et al. [90], the ability to derive meaning through work can bolster an individual’s capacity to cope with challenges and setbacks. Those who find meaning in their work are more likely to view difficulties as opportunities for growth rather than insurmountable obstacles.

Lutgen-Sandvik et al. [91] elucidate that engaging in meaning-making through work can evoke positive emotions such as joy, satisfaction, and pride. Experiencing these positive emotions in the context of one’s work contributes to an overall positive mental state. Rothausen and Henderson [92] identified that meaningful work is associated with higher levels of overall well-being. When individuals perceive their work as meaningful, it positively influences various dimensions of well-being, including emotional, social, and psychological well-being. Previous studies indicate that meaning-making through work is a vital component of meaningful work, playing a crucial role in promoting mental health.

Previous studies show that organizations that cultivate a culture where employees can derive meaning from their work contribute to a positive and supportive work environment, yielding lasting benefits for the mental well-being of their workforce. Therefore, we posit the following hypotheses:

• *Hypothesis 1b Meaning making through work is significantly related to meaningful work.*

#### Greater good motivations

Greater good motivations constitute a crucial element of meaningful work [55]. As highlighted by Nikolova and Cnossen [93], when individuals are driven by a desire to contribute to the greater good or make a positive impact on others and society, it imbues their work with added significance and purpose. Allan et al. [84] elucidate that working towards the greater good provides individuals with a sense of purpose that transcends personal or financial goals, thereby enriching the overall meaningfulness of the work.

According to Onça and Bido [94], motivations centered around the greater good often align with intrinsic motivation, where individuals are propelled by a genuine interest in making a positive impact. Intrinsic motivation is associated with increased job satisfaction and a more positive mental outlook. Jiang and Johnson [60] assert that contributing to the greater good and assisting others can result in a sense of altruistic satisfaction. Knowing that one’s work positively influences others can evoke feelings of fulfillment and contentment.

Ghadi et al. [95] suggest that engaging in work that benefits the greater good can contribute to higher levels of overall well-being. The awareness that one is making a positive contribution to society or a community can foster a more positive emotional state. Tan et al. [96] propose that pursuing greater good motivations can lead to a more enduring and sustainable sense of satisfaction, contributing to a stable and positive mental state. Greater good motivations emerge as a potent component of meaningful work, significantly enhancing mental health. Individuals who find purpose and meaning in contributing to a cause beyond themselves often experience a deeper sense of fulfillment and satisfaction, thereby positively impacting their overall mental well-being. Therefore, we posit the following hypotheses:

• *Hypothesis 1c. Greater good motivations is significantly related to meaningful work.*

The identity and well-being of individuals are significantly influenced by their employment; however, much of the research often focuses on the mere presence or absence of work rather than delving into the subjective significance of work to individuals [83]. While numerous studies have established links between a general sense of meaning in life and factors such as job satisfaction [e.g., 97; 98] and health-related behaviors [e.g., 99; 100], there has been less emphasis on the concept of meaningful work.

Meaningful work, as described by Steger et al. [55], is characterized by an individual perceiving their work as personally significant and contributing to a greater societal benefit. Research has shown that meaningful work can have substantial advantages for both individuals and organizations, particularly in terms of the mental well-being of employees [101]. This highlights the significance of not just having a job but also perceiving one’s work as meaningful and purposeful. Such a perspective can result in favorable outcomes, benefiting both the individuals themselves and the organizations they are affiliated with.

Despite the evident importance of meaningful work for enhancing worker happiness and generating positive organizational outcomes [as demonstrated by studies like 58 and 102], there has been limited exploration of the connection between meaningful work and its impact on health. Consequently, we would like to highlight research indicating that meaningful work has a positive influence on employees’ mental health, especially during challenging times such as the COVID-19 pandemic.

Furthermore, Ryff and Singer [103] have advocated for increased research into “how employment helps individuals discover meaning in their lives, realize their true selves, and utilize their unique talents, ultimately leading to improved health” (p. 8). This underscores the need for a deeper understanding of how work can contribute to individuals’ sense of purpose and well-being, ultimately leading to better overall health outcomes.

The study of Allan et al. [83] has demonstrated that meaningful work can mitigate the effects of job stress and improve the mental health of employees. This is particularly relevant during crisis situations in emerging markets. A qualitative study conducted by Mousa and Samara [8] revealed that in the context of the COVID-19 pandemic, employees who found their work to be meaningful reported experiencing lower levels of stress and better mental health.

Considering the characteristics of SME employees, who often contend with heavy workloads [104], overtime demands, and various administrative responsibilities, participants in the study expressed that their job roles contributed to persistent feelings of depression, anxiety, stress, and other mental health issues [105], even amid the pandemic. While meaningful work seems to act as a protective factor, there has been no research exploring whether it can mitigate the impact of stress and enhance the mental health of SME employees during the COVID-19 pandemic. As a result, this study proposes the following hypotheses to investigate the potential influence of meaningful work on the mental health of SME employees:

• *Hypothesis 1 Meaningful work has a positive effect on the mental health of SME employees during COVID-19 pandemic in Malaysia.*

### Mediating effect of coping strategies

Coping is reacting upon a cognitive assessment that something is putting a strain on a person’s capabilities, like loss, challenge, or danger [75]. According to Lazarus and Folkman [43], individuals undergo the primary appraisal process when confronted with such a challenge, wherein they ask themselves when a scenario would affect their mental health. This is accompanied by the supplementary assessment process, wherein people assess their own ability to successfully handle the source of stress [40]. Problem-focused coping involves using tactics to directly address the source of stress; it is more prevalent when people feel they can control a stressor [74]. Active coping, according to Carver et al. [106], is at the heart of problem-focused coping, as defined by Lazarus and Folkman [43]. Acting coping includes taking some action to alleviate a source of stress, planning actions to improve mental health, and putting aside unproductive activities in order to focus less on the challenges [106]. Problem-focused coping styles are frequently linked to favorable psychological outcomes [107]. Such coping techniques have been connected to vocational psychology, which has been shown to promote mental health by focusing on meaningful work [108]. Action coping, as explained by Lazarus and Folkman [43], has been shown to have a key mediating role in the interaction between people and their environments. When making efforts to manage a person’s stress and improve mental health efficiently [109], one should concentrate on meaningful work, particularly in a crisis like the COVID-19 pandemic [110]. Previous studies concentrating on COVID-19 [such as, 111; 112], have shown that problem-focused coping strategies are beneficial in lowering psychological symptoms in frontline healthcare workers. In vocational psychology, problem-focused coping can also provide an individual with a degree of stability over the source of stress [113], which is a key predictor of psychological well-being and meaningful work. Furthermore, the mediating effect of problem-focused coping among meaningful work and mental health has received insufficient attention, particularly from the perspective of SME employees in the COVID-19 pandemic.

Carver et al. [106] stated that “passive coping is an emotion-focused coping strategy, behavioral detachment (stop attempting to handle a stressor), mental detachment (stop thinking about the objective the stressor is related to), and rejection (pretend that the stressor does not exist) are all examples of passive coping” (p. 34). Passive coping is usually related to vocational psychological aspects such as employee well-being and meaningful activities. An emotion-focused form of coping, according to Lazarus and Folkman [43], improves a person’s mental health by allowing them to do meaningful work. Lorente et al. [114] found that emotion-focused interventions minimize psychological discomfort among healthcare workers. Furthermore, emotion-focused coping has a positive association with meaningful work to prevent hazardous situations in the workplace [115]. Hence, when faced with a critical situation such as a pandemic, emotion-focused coping behaviors are capable of interfering with more effective forms of coping, resulting in improved mental health and employees more focused on meaningful work. Furthermore, the role of emotion-focused coping in mediating the association between meaningful work and mental health has received less attention, particularly from the perspective of SME employees affected by the COVID-19 epidemic. Therefore, we propose the following:

• *Hypothesis 2 Meaningful work has a positive relationship with problem-focused coping.*

• *Hypothesis 3 Problem-focused coping has a positive relationship with mental health of SME employees.*

• *Hypothesis 4 Meaningful work has a positive relationship with emotion-focused coping.*

• *Hypothesis 5 Emotion-focused coping has a positive relationship with mental health of SME employees.*

• *Hypothesis 6 The mediating effect of problem-focused coping between meaningful work and the mental health of SME employees during COVID-19 pandemic in Malaysia.*

• *Hypothesis 7 The mediating effect of emotion-focused coping between meaningful work and the mental health of SME employees during COVID-19 pandemic in Malaysia.*

### Hypothesized framework

**Fig. 1:**
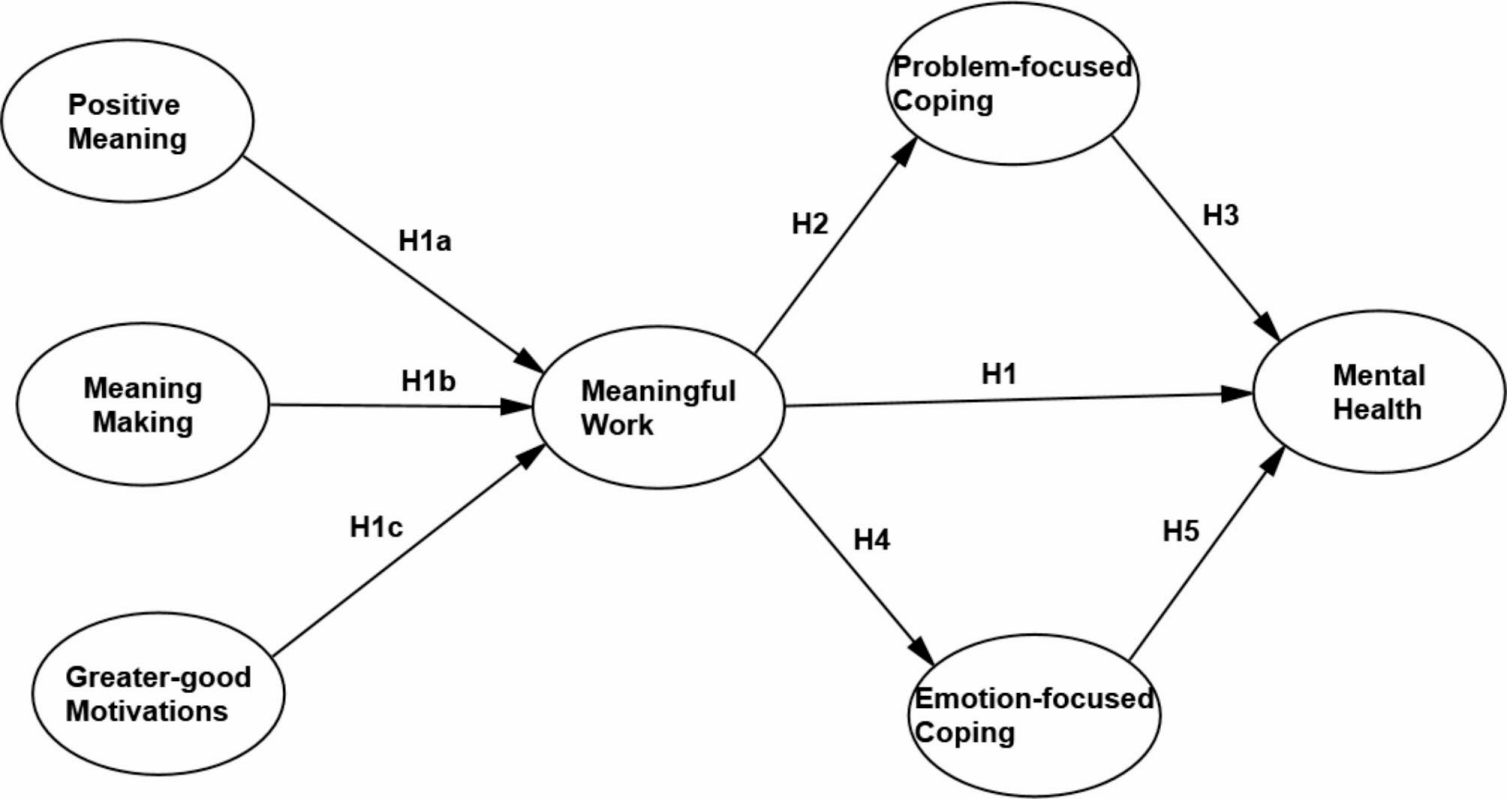
Hypothesized framework

This study contributes to the literature by exploring the impact of meaningful work on the mental health of SME employees, particularly in the context of the COVID-19 pandemic, and by examining the mediating role of coping strategies. The research objectives include identifying the components of meaningful work, assessing its direct influence on SME employees’ mental health, and evaluating the mediating effects of both problem-focused and emotion-focused coping. The hypothesized framework, illustrated in Fig. 1, guides the investigation and enhances our understanding of the dynamics between meaningful work, coping strategies, and mental health in the context of SMEs during the pandemic.

## Methods

### Data collection: procedure and sample

This research employed a quantitative approach to assess the hypotheses and achieve the research objectives. Due to the constraints imposed by the COVID-19 pandemic, data collection was conducted using a self-administered questionnaire approach and distributed online. The questionnaires were administered by the researchers who either traveled to the respondents’ locations or sent the questionnaires via email. The data were collected from December, 2021 to July, 2022.

The researchers took measures to ensure that respondents’ responses would be kept confidential and anonymous. By providing this assurance, it aimed to create an environment in which respondents felt that their responses were private and not linked to their identities. This approach is crucial because when respondents believe that their responses are kept confidential and anonymous, they are more likely to provide honest and candid answers, rather than feeling pressured to conform to perceived expectations or provide socially desirable responses. This, in turn, enhances the quality and accuracy of the collected data.

In this study, the final sample comprised non-English speakers, necessitating a process of translation and back-translation of the research instrument. This translation process occurred in two stages: In the first stage, following the creation of the original questionnaire in English, it was translated into Malay by a qualified translator who was proficient in both Bahasa Malay and was a native Malaysian speaker. Subsequently, in the second stage, another qualified translator, whose native language was English, performed a back-translation of the Malay version. This back-translation was carried out to confirm the equivalence of the questionnaire translations and to make necessary adjustments to address any variations or discrepancies that arose during the translation process. This meticulous process of translation and back-translation helps ensure the accuracy and fidelity of the questionnaire when administered to non-English-speaking participants, helping to maintain the integrity of the research instrument across different language versions.

In this study, the participants were employees of Small and Medium Enterprises (SMEs) located in various cities across Malaysia. To ensure a representative sample, the researchers employed a stratified random sampling method. We selected participants from a pool of 213 SMEs listed by SME Corp Malaysia. According to Musa and Chinniah [116], the services and manufacturing sectors dominate the Malaysian SME landscape, comprising 90.6% of all SMEs and contributing significantly to the country’s overall GDP (83.3%).

Given this, the study focused on two distinct strata of SMEs: the manufacturing sector and the service sector. According to data from the SME Corp Malaysia Annual Report 2022, the approximate number of employees in the service sector was around 5,000, while the manufacturing sector had approximately 3,000 employees. To determine the appropriate sample sizes for each stratum, the researchers followed Krejcie and Morgan’s [117] criteria. This led to a sample size of 357 for the service sector and 341 for the manufacturing sector.

In total, the researchers distributed 698 questionnaires to potential respondents. However, only 462 surveys were completed, resulting in a response rate of 66.2%. This response rate indicates the proportion of participants who provided usable responses out of the total number of questionnaires distributed.

### Measurement of variables

Hair et al. [118] describe the survey questionnaire as a carefully designed instrument tailored to gather the necessary information to address the research inquiries and fulfill the principal objectives of the study. The data collection for this study involved the use of adapted questionnaire items drawn from prior research, with the aim of assessing the impact of meaningful work on the mental health of SME employees through their coping strategies.

To facilitate participant engagement and comprehension, the questionnaire featured items rated on a 7-point Likert scale, allowing respondents to provide nuanced responses. Specifically: Items measuring meaningful work, including aspects such as positive meaning in work, meaning making through work, and greater good motivations, were adapted from Steger et al. [55]. To assess mental health, questionnaire items were adapted from Hu et al. [119] and Mazaherinezhad et al. [120]. For the evaluation of coping strategies, specifically problem-focused coping and emotion-focused coping, items were adapted from Jalil et al. [40]. This careful adaptation of items from established studies ensured that the questionnaire was not only comprehensive but also built upon established measurement approaches in the relevant research areas.

### Ethical consideration

The research procedure received approval from the Universiti Malaysia Sarawak Ethics Committee (UNIMAS-EC), in accordance with the principles outlined in the Declaration of Helsinki. All participants in the study provided written consent before their data was collected. Subsequently, the gathered data was subjected to further analysis as part of the research process.

### Statistical analysis

The analysis of the data in this study employed structural equation modeling (SEM) to assess the proposed hypotheses. A maximum likelihood approach within SEM was employed to evaluate both the structural and measurement models.

To assess the convergent validity and the causal relationships among the modified items and variables within the measurement model, confirmatory factor analysis (CFA) was conducted. Subsequently, a structural model was employed to investigate the connections between exogenous (independent) and endogenous (dependent) factors. For the SEM analysis, the researchers utilized the AMOS 21.0 software tool. It’s worth noting that, as per Kline’s [121] recommendations, an acceptable model typically requires a minimum sample size of 100 to 200 respondents. This comprehensive approach allowed for the rigorous examination of the research hypotheses and provided a sound basis for drawing conclusions from the data.

## Results

### Demographic characteristics

Demographic surveys serve the purpose of providing essential information about participants, aiding researchers in understanding where individuals fit within the broader population [122].

In the context of this study, demographic data was collected from a total of 462 employees employed across 213 randomly selected SMEs. Table 1 in this research provides a comprehensive overview of the demographic information of the surveyed individuals. This information is valuable for understanding the characteristics of the study participants and their context, which is essential for analyzing the research findings in a more meaningful way.

**Table 1:** Demographic profile of respondents

Constructs		Number	Percentage
Gender	Male	297	64.3%
	Female	165	35.7%
Age	Below 25	36	7.8%
	25–35	117	25.3%
	36–45	126	27.3%
	46–55	158	34.2%
	55 and above	25	5.4%
Marital status	Single	77	16.7%
	Married	284	61.5%
	Widow	46	9.9%
	Divorced	55	11.9%
Education	Diploma or high school or less	159	34.4%
	Bachelors	188	40.7%
	Masters	94	20.4%
	Doctorate	21	4.5%
Ethnic group	Malay	196	42.2%
	Chinese	153	33.2%
	Indians	75	16.3%
	Others	38	8.3%
Religion	Muslim	213	46.1%
	Hindu	69	14.9%
	Christian	94	20.3%
	Buddhist	74	16.1%
	Others	12	2.6%
Enterprises level (based on number of employees, N = 213)	Small enterprise	134	62.9%
	Medium enterprise	79	37.1%
Enterprises activities (N = 213)	Manufacturing sector	55	25.8%
	Services sector	158	74.2%
Position in the enterprise	Lower level executives	97	21.0%
	Middle level executives	236	51.1%
	Upper level executives	129	27.9%
Income level	Less than RM3000	104	22.5%
	RM3000-4000	238	51.5%
	RM4001-5000	81	17.5%
	RM 5001 and above	39	8.5%

Note: N = number of selected SMEs

### Normality statistics

Yun et al. [123] conducted tests for single-variable and multivariate normal distribution before proceeding with the structural equation modeling procedure. Tabachnick and Fidell [124] clarify that, in the assessment of univariate normality, skewness and kurtosis values are expected to fall within the range of + 1 to -1. Following the analyses, it was established that the dataset satisfied the assumptions of univariate normality. According to Kline [121], multivariate normality analyses were then carried out to ascertain whether the dataset exhibited a multivariate normal distribution. The results of the final analyses, presented in Table 2, demonstrate that the data utilized in the study conformed to a reasonably normal distribution. The dataset met the criteria for multivariate normality assumptions. In this stage, cases with a multivariate kurtosis value within the range of + 2 to -2, and a multivariate critical ratio value less than 1.96, were considered as meeting the established criteria [121].

**Table 2:** Normality assessment

Variables	Likert scale	Mean	min	max	Skewness	c.r.	Kurtosis	c.r.
PM	1–7	5.54	12.000	38.000	0.072	1.032	0.228	2.247
MM	1–7	5.97	16.000	44.000	0.327	3.867	0.084	1.336
GM	1–7	6.21	21.000	52.000	0.091	1.053	0.457	4.289
PFC	1–7	5.11	18.000	46.000	0.309	3.271	0.298	2.731
EFC	1–7	4.98	22.000	58.000	-0.037	-1.052	0.163	2.019
MH	1–7	5.36	19.000	55.000	0.644	4.336	-0.081	-1.021
Multivariate							0.364	0.632

Note: PM (positive meaning), MM (meaning making), GM (greater good motivations), PFC (problem-focused coping), EFC (emotion-focused coping), MH (mental health)

### Analysis of measurement model

During the assessment of the overall measurement model, the researchers used modification indices to identify potential issues. These indices indicated that certain indicators, specifically PM_5 (Positive meaning), MM_4 (Meaning making), GM_4 (Greater good motivations), and PFC_5 (Problem-focused coping), had unacceptably high values, suggesting redundancy or overlap in these items. To address this, an iterative process was undertaken to remove these redundant items.

**Fig. 2:**
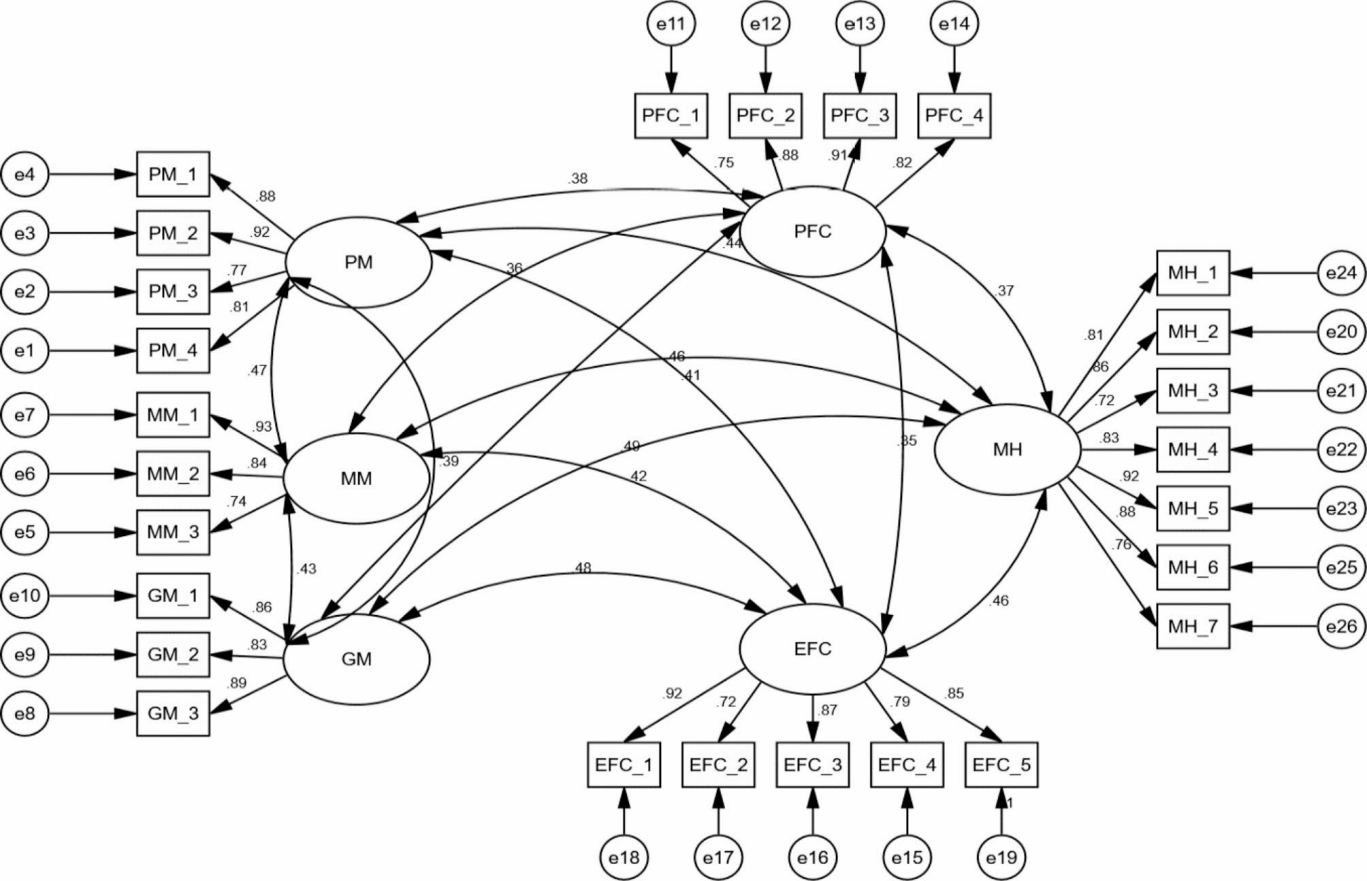
Measurement model

Following the removal of these items, the overall model fitness improved and met the desired criteria. As depicted in Fig. 2, the measurement model demonstrates that each item’s factor loading is deemed adequate, surpassing the threshold of 0.70, as recommended by Song et al. [125]. This indicates that the remaining items are effective indicators of their respective constructs, enhancing the reliability and validity of the measurement model.

To estimate the parameters of the model, the researchers employed the maximum-likelihood technique, with all analyses relying on variance-covariance matrices. In assessing the goodness-of-fit of the model, it’s essential to consider various fit indices, as recommended by Hair Jr. et al. [126]. These fit indices help gauge how well the model aligns with the observed data and provide insights into its overall adequacy and accuracy. The model goodness-of-fit in this research was in the acceptable range (RMSEA = 0.039; chi square = 589.849; df = 461; GFI = 0.922; AGFI = 0.936; CFI = 0.941; CMIN/df = 1.422).

Hair Jr. et al. [126] assert that validity and reliability can be assessed using metrics such as “Composite Reliability (CR) and Average Variance Extracted (AVE).“ They suggest that CR should ideally exceed 0.7 to demonstrate reliability and, at a minimum, should be above 0.6. Additionally, AVE should surpass 0.5 to establish convergent validity.

In the context of this study, all variables exhibited AVE values exceeding 0.5, and CR values surpassing 0.7, as presented in Table 3. These results indicate that all components within the model demonstrate strong reliability and convergent validity, further bolstering the robustness of the study’s findings.

**Table 3:** AVE and CR evaluation

Items	Measurement Path	FL	CR	AVE
**Positive meaning**				
PM_1	I have a meaningful career in the COVID-19 pandemic.	0.88	0.91	0.72
PM_2	In the context of the pandemic, I understand how my work adds to the meaning of my life.	0.92		
PM_3	In a crisis, I have such a strong understanding of what makes my work meaningful.	0.77		
PM_4	I’m working on a project in pandemic that I enjoy.	0.81		
**Meaning making**				
MM_1	In the COVID-19 pandemic, I view my work as contributing to my own progress.	0.93	0.88	0.71
MM_2	My work assists in my understanding of myself during a pandemic.	0.84		
MM_3	During a pandemic, my work helps me make sense of the world around me.	0.74		
**Greater good motivations**				
GM_1	My work has a significant impact on the organization during the COVID-19 pandemic.	0.86	0.89	0.74
GM_2	During a pandemic, I know my work has a good impact on the organization.	0.83		
GM_3	In times of crisis, the work I undertake has a larger meaning.	0.89		
**Problem-focused coping**				
PFC_1	Make use of my prior experience; I’ve been in a similar situation.	0.75	0.91	0.71
PFC_2	Come up with a few potential solutions to the issue.	0.88		
PFC_3	I try to figure out what’s going on in order to have a better perspective of the scenario.	0.91		
PFC_4	I’ve developed a strategy and am sticking to it.	0.82		
**Emotion-focused coping**				
EFC_1	I tried not to become emotional when I thought about or was reminded of the present problem.	0.92	0.92	0.69
EFC_2	I stay away from anyone or anything that reminds me of the current situation.	0.72		
EFC_3	I was filled with a swarm of strong feelings regarding the current scenario.	0.87		
EFC_4	I’m attempting to focus on finding a solution to the current problem.	0.79		
EFC_5	I tried to forget about the current situation and focus on future recovery.	0.85		
**Mental health**				
MH_1	During a crisis, I am able to focus on my work.	0.81	0.94	0.69
MH_2	I did not lose much sleep because of anxiety during the COVID-19 pandemic.	0.86		
MH_3	During the recent crises, I did not feel overworked.	0.72		
MH_4	I am optimistic that I will be able to tackle my work-related challenges during the pandemic.	0.83		
MH_5	I do not feel unhappy or depressed.	0.92		
MH_6	Recently, I’ve begun to believe in myself.	0.88		
MH_7	In pandemic, I consider myself to be a valuable individual.	0.76		

### Assessment of structural model

Following the assessment of Composite Reliability (CR) and Average Variance Extracted (AVE), Boker et al. [127] recommend investigating the relationships between exogenous (independent) and endogenous (dependent) latent constructs. This examination typically takes place at the structural model stage. Additionally, as emphasized by Iasiello et al. [128], it’s crucial to evaluate the goodness-of-fit for the structural model.

**Fig. 3:**
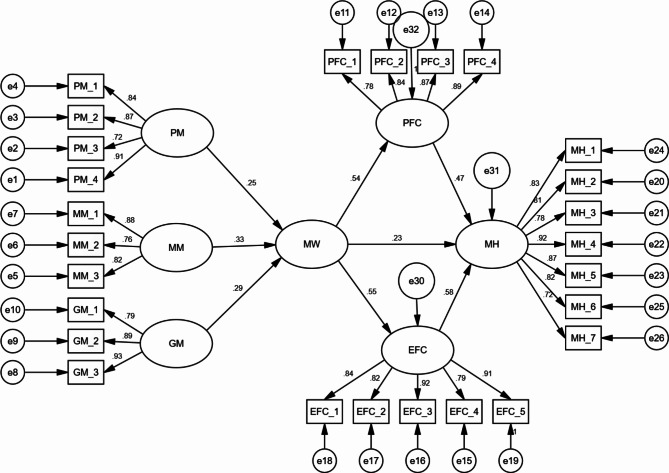
Structural model

The study’s findings indicate that the model exhibits a well-fitted structure, as evidenced by the fit indices (Chi-square = 1.215; GFI = 0.942; AGFI = 0.936; CFI = 0.954; TLI = 0.959; RMSEA = 0.048), as illustrated in Fig. 3. These indices collectively indicate that the model aligns well with the observed data and provides a strong basis for interpreting the study’s results.

The subsequent phase in this research involves scrutinizing the hypothesized relationships within the model. Specifically, hypotheses denoted as “H1a, H1b, H1c, H1, H2, H3, H4, and H5,“ as outlined in Table 4, were found to be statistically significant. This signifies that the study’s proposed research model effectively supports and validates these hypothesized links, lending credibility to the study’s findings.

**Table 4:** Testing direct relationship

Hypothese and Paths	ß	Z-Value	Supported
H1a: Positive meaning Meaningful work	0.25**	3.427	Yes
H1b: Meaning making Meaningful work	0.33***	4.684	Yes
H1c: Greater good motivations Meaningful work	0.29***	3.879	Yes
H1: Meaningful work Mental health	0.23**	3.363	Yes
H2: Meaningful work Problem-focused coping	0.54***	5.764	Yes
H3: Problem-focused coping Mental health	0.47***	5.107	Yes
H4: Meaningful work Emotion-focused coping	0.55***	5.973	Yes
H5: Emotion-focused coping Mental health	0.58***	6.014	Yes

*p < 0.05, **p < 0.01, ***p < 0.001

### The mediation analysis

Hypotheses 6 and 7 in this study were designed to explore whether problem-focused and emotion-focused behaviors mediate the relationship between meaningful work and mental health among SME employees. As per Awang et al. [129], “partial mediation occurs when the indirect effect is greater than the direct effect between the variables and the direct effect is also substantial.“

The study’s findings indicate that the indirect effect of problem-focused work on mental health amounted to 0.25 (calculated as 0.54 × 0.47 = 0.25), while the direct effect was 0.23. Similarly, the indirect effect of emotion-focused work on mental health was 0.32 (calculated as 0.55 × 0.58 = 0.32), while the direct effect was 0.25. These results suggest that problem-focused and emotion-focused behaviors partially mediate the relationship between meaningful work and mental health in SME employees, as the indirect effects are greater than the direct effects, and the direct effects are substantial.

In this study, it’s important to note that the results of any mediation test were further verified using the bootstrapping procedure, as recommended by Awang et al. [129]. To conduct this verification, the researchers employed the Maximum Likelihood Bootstrapping procedure with a bootstrap sample size of 1000 and a bias correction confidence interval of 95%. The results of this bootstrapping procedure are presented in Table 5, providing additional confirmation and robustness to the mediation findings.

**Table 5:** Bootstrapping results

Paths	Relationship	Mediation
Meaningful work Mental health	0.23	
Meaningful work Problem-focused Mental health	0.54 x 0.47 = 0.25	Partial
Meaningful work Emotion-focused Mental health	0.55 x 0.58 = 0.32	Partial

## Discussion and implications

### Discussion

The nature of SME employees’ jobs, which often involves managing a work-from-home schedule, working long hours without job security, and handling various clerical tasks, has exposed them to ongoing challenges. These challenges have, in many cases, led to experiences of anxiety, depression, stress, and other psychological health issues during the COVID-19 pandemic.

Work plays a crucial role and has a profound influence on an employee’s psychological well-being [8]. Researchers and SME managers/owners, aiming to enhance employee performance, job satisfaction, and overall enterprise success, are increasingly exploring the relationship between perceptions of meaningful work and mental health. Consequently, this study makes valuable contributions to areas where existing literature remains inconclusive. First, it identifies the components that constitute meaningful work. Second, it highlights the significant connection between meaningful work and the mental health of SME employees during the COVID-19 pandemic. Third, this research delves into the mediating role of coping strategies in the relationship between meaningful work and the mental health of SME employees during the pandemic. These contributions enrich our understanding of how work experiences and coping mechanisms can impact the well-being of SME employees in challenging times.

Indeed, despite the relatively limited prior research on meaningful work, its significance and potential for further study are evident. This research has advanced our understanding of meaningful work by identifying its components, which include positive meaning, meaning making, and greater good motivation. These components hold important implications for vocational psychology, shedding light on how they contribute to employees’ well-being and job satisfaction. These findings align with previous research by Steger et al. [55], highlighting the substantial impact of meaningful work dimensions on psychological well-being. The recognition of the significance of all three dimensions underscores the practical utility of describing each dimension separately. This granularity can be valuable in real-world applications, helping individuals and organizations pinpoint specific aspects of job satisfaction and areas for improvement.

The primary objective of this research was to investigate whether meaningful work predicts the mental health of SME employees during the COVID-19 pandemic. Given the context of the pandemic, it was anticipated that meaningful work would be associated with employee mental health. The study confirmed that meaningful work indeed enhances the mental health of employees, thereby substantiating our initial hypotheses. These findings align with recent research conducted by Mousa and Samara [8], which also suggests the positive impact of meaningful work on mental health.

Folkman and Lazarus [130] have emphasized the significant mediating role of coping in the individual-environment relationship, particularly the roles of problem-focused and emotion-focused coping strategies. This study’s findings have confirmed the partial mediation effect of problem-focused and emotion-focused coping on the relationship between meaningful work and the mental health of SME employees during the pandemic. The research suggests that SME owners and managers can promote meaningful work and enhance the mental health of their employees by encouraging problem-focused and emotion-focused coping strategies. These findings align with previous research by Lee et al. [131], highlighting the indirect influence of coping strategies on employees, alongside the domain of vocational psychology.

### Theoretical implications

The first significant contribution of this study pertains to the examination of how meaningful work impacts the mental health of SME employees during the COVID-19 pandemic. While Mousa and Samara [8] conducted a qualitative analysis and found a statistically significant association between meaningful work and mental health, this study opted for a quantitative research approach employing a survey questionnaire to gather comprehensive insights into the underlying mechanisms of this phenomenon, particularly within the framework of the self-determination theory. Upon thorough data analysis, the study’s authors concluded that meaningful work among SME employees in Malaysia can indeed enhance mental health conditions. In light of these findings, it is suggested that providing SME workers with meaningful work can potentially contribute to a reduction in mental health issues.

The second noteworthy theoretical contribution of this study lies in its empirical demonstration of how coping strategies mediate the relationship between meaningful work and the mental health of SME employees. Prior research did not explore the intricate link between these factors. Therefore, this study fills a crucial gap by providing empirical evidence of how SME employees can bolster their mental health and mitigate the impacts of COVID-19 through the utilization of problem-focused and emotion-focused coping strategies, which are facilitated by their engagement in meaningful work. The findings reveal that meaningful work plays a crucial role in enhancing mental health and in enabling SME employees to navigate and overcome challenges posed by disasters such as the COVID-19 pandemic, while also fostering the adoption of coping strategies to fortify this relationship.

The third significant theoretical contribution of this study is its empirical validation of the importance of meaningful work (including positive meaning, meaning making, and greater good motivation) within the framework of self-determination theory, especially in the context of a pandemic. The study demonstrates that SME employees should focus on their meaningful work during a pandemic to maintain a sense of purpose in their responsibilities, ultimately alleviating potential mental health issues. This finding opens the door for further research and encourages other scholars to reevaluate self-determination theory and explore its applicability in non-crisis situations. It highlights the enduring relevance of self-determination theory and its potential to enhance well-being, not only during crises but also in everyday circumstances.

### Practical implications

First, this study proposes that the concerned SME owners put pressure on their managers to execute a business strategy that guarantees mental health issues are mitigated. Such initiatives are not limited to the Malaysian setting but also apply to other emerging economies and comparable institutional systems where mental health is underserved. Securing SME employees’ access to mental health facilities and strategies for coping with mental illnesses should be part of the strategy plan, as should hiring qualified instructors to control the mental health challenges faced by employees, in addition to developing preventive measures such as increasing meaningful work and adopting coping strategies.

Second, this research recommends that SME employees see a psychiatrist on a regular basis to assess their depression and anxiety levels. This would help them prevent any health issues that could arise as a result of their inability to focus on meaningful work. Moreover, the expansion of the COVID-19 pandemic and the work from home should not be seen as having a long-term purpose. Employees in small businesses should acquire coping strategies to help them cope with depression and anxiety.

Third, we recommend that SME employees’ draught a report in which each employee details the issues he or she is having with coworkers and managers/owners, as well as solutions that might help them maintain their positive attitude. Further, we suggest that employees, rather than managers, should discuss the important concerns identified by these reports in meetings with stakeholders. This helps in offering significant social support to employees, as well as demonstrations of gratitude and significance.

## Limitations and suggestions for future research

The main limitation of this research is that it focuses solely on the perspectives of SME employees without taking into account the perspectives of their managers or owners. Addressing employees in manufacturing and service sector SMEs alone, while ignoring those in other sectors (such as agriculture and construction), and may also be seen as a limitation. Both the first and second limitations, according to the researchers, may limit their capacity to generalize the findings of the study. The cross-sectional method, on the other hand, has limitations when it comes to studying the causal linkages between meaningful work, coping techniques, and mental health.

Future research endeavors could delve into the experiences and insights of SME owners and managers, shedding light on the challenges they face and the strategies they employ to mitigate anxiety, depression, and other mental health issues, particularly in the post-COVID-19 era. Additionally, the authors of this study encourage collaboration among scholars in the realm of vocational psychology to engage in interdisciplinary scholarly endeavors aimed at identifying the key factors that can have a positive impact on employees’ mental health. Such endeavors can contribute to a comprehensive understanding of the complex dynamics between work, mental health, and well-being.

Finally, the authors suggest other researchers perform longitudinal studies in order to identify precise causal relationships between meaningful work and mental health before and after a crisis. Furthermore, our research focused on Malaysian SME employees. Bringing this approach to different market contexts can give further insight into the environmental and institutional elements at play in each developing or developed country and how they impact employee mental health, particularly during crisis situations.

## Data Availability

All the data generated or analyzed during this study are included in this published article and available from the corresponding author on reasonable request.
